# Prognostic value of adjuvant external beam radiotherapy for papillary thyroid cancer based on competitive risk model and propensity score matching

**DOI:** 10.1038/s41598-023-34269-7

**Published:** 2023-04-28

**Authors:** Jiani Zhou, Chaoqun Wu, Shihong Fan, Miaohui Zhao

**Affiliations:** 1 Department of General Practice, Ningbo Medical Treatment Center Li Huili Hospital, No. 57, Xing Ning Road, Yinzhou District, Ningbo, 315040 People’s Republic of China; 2 Department of Endocrinology Department, Ningbo Medical Treatment Center Li Huili Hospital, Ningbo, 315040 People’s Republic of China

**Keywords:** Endocrinology, Oncology

## Abstract

This study aimed to assess the impact of adjuvant external beam radiotherapy (EBRT) on the survival of patients with locally invasive papillary thyroid carcinoma. This retrospective study used data from the Surveillance, Epidemiology, and End Results database for the diagnosis of papillary thyroid carcinoma, using Cox models to screen for adverse prognostic factors. The prognostic value of using adjuvant external beam radiotherapy in papillary thyroid carcinoma was further evaluated, based on the competing risk model and propensity score matching. Based on the competitive risk model, the sub-distribution hazard ratio (SHR) of the multivariate analysis of patients receiving EBRT alone versus those receiving radioiodine-131 alone was 9.301 (95% CI 5.99–14.44) (P < 0.001), and the SHR of the univariate analysis was 1.97 (95% CI 1.03–3.78) (P = 0.042). In the propensity score-matched Kaplan–Meier analysis, patients who received EBRT still had worse OS (6-year OS, 59.62% vs 74.6%; P < 0.001) and DSS (6-year DSS, 66.6% vs 78.2%; P < 0.001) than patients who did not receive EBRT. Patients who received EBRT had a higher cumulative risk of death due to thyroid cancer after PSM (P < 0.001). Adjuvant EBRT was not associated with survival benefit in the initial management of locally invasive papillary thyroid cancer.

## Introduction

Thyroid cancer is the most common malignancy of the endocrine system^1,2^. Recently, due to improvements in imaging and molecular pathology detection technology for tiny papillary thyroid cancer, the detection rate of thyroid cancer has increased by 10% or higher annually, and that of middle and advanced thyroid cancers has also shown an upward trend^3–5^. The standard treatment for patients with well-differentiated thyroid cancer is total thyroidectomy, and patients usually receive adjuvant radioiodine-131 (RAI) to ablate the residual thyroid and treat residual iodine-dependent disease^6,7^.

The results of published studies on the use of external beam radiotherapy (EBRT) in papillary thyroid carcinoma (PTC) are inconsistent. Some authors did not find statistically significant improvements in survival but documented adverse effects of EBRT, therefore, it was not recommended^8–10^. Other studies have shown impressive improvements in survival outcomes and local control in the absence of major complications and recommended its use in specific settings^11–15^. The American Thyroid Association guidelines state that, after complete surgical resection, routine neck adjuvant EBRT had no effect. They recommend combining RAI and EBRT in patients with disease involving the upper aerodigestive tract^16^.

Despite the increasing incidence of well-differentiated thyroid cancer, there has been little success in conducting randomized controlled trials to assess the benefit of EBRT. A multicenter study was conducted in Germany that randomized patients with pT4 disease to either an EBRT group or non-EBRT group after thyroidectomy, RAI therapy, and TSH inhibition. Only 45 of 311 patients agreed to randomization, and the study design was changed to a prospective cohort study. The authors concluded that routine EBRT does not recommend treatment for pT4 disease. However, this study has some limitations, the most important limitation being the lack of sufficient number of randomized patients and the large amount of crossover to the non-irradiated group of the trial, in addition to limited follow-up time (mean 930 days) and potential underreporting of events^8^. This led us to obtain a better summary of the available data to further evaluate the prognostic value of EBRT for locally invasive thyroid cancer in adults.

This study aimed to include patients with locally invasive thyroid cancer undergoing partial or subtotal resection through the Surveillance, Epidemiology and End Results database. Explore the prognostic value of EBRT based on competitive risk models, and control the influence of confounders on the conclusions by propensity score matching (PSM).

## Results

### Survival analysis

**Figure 1:**
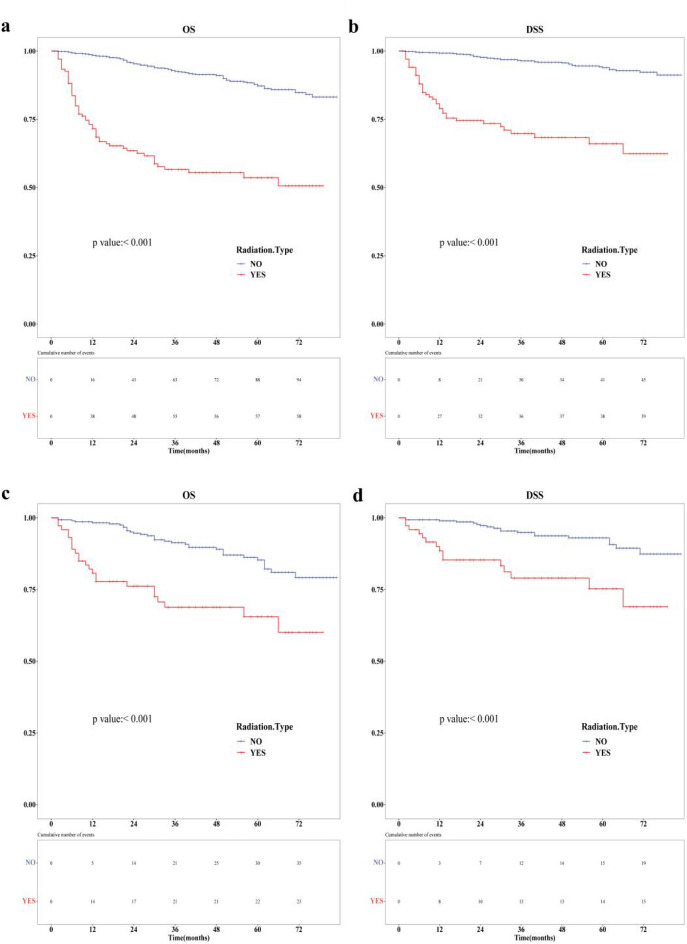
(**a**) Before propensity score-matched Kaplan–Meier analysis: overall survival as a function of EBRT. (**b**) Before propensity score-matched Kaplan–Meier analysis: disease-specific survival as a function of EBRT. (**c**) After propensity score-matched Kaplan–Meier analysis: overall survival as a function of EBRT. (**d**) After propensity score-matched Kaplan–Meier analysis: disease-specific survival as a function of EBRT.

The SEER database identified 1181 patients who met the inclusion criteria. Of these, 135 patients received EBRT. The comparison of patient characteristics between the two treatment groups (before and after PSM) is shown in Table 1. Male patients, older patients, and patients with AJCC stage IV, AJCC T4b stage, AJCC M1 stage, grade III–IV stage, with chemotherapy, tumor size > 30 mm, and total thyroidectomy were more likely to be treated with EBRT. Unadjusted Kaplan–Meier analysis showed that patients who received EBRT had worse OS (6-year OS, 50.6% vs 76.2%; P < 0.001) (Fig. 1a) and DSS (6-year DSS, 59.4% vs 79.6%; P < 0.001) (Fig. 1b) than patients who did not receive EBRT.

**Table 1:** Patient characteristics before PSM.

Variables	Total (n = 1181)	No (n = 1046)	Yes (n = 135)	p
Age.cat, n (%)				< 0.001
~ 50	458 (39)	434 (41)	24 (18)	
51–65	377 (32)	333 (32)	44 (33)	
66~	346 (29)	279 (27)	67 (50)	
Sex, n (%)				0.004
Female	765 (65)	693 (66)	72 (53)	
Male	416 (35)	353 (34)	63 (47)	
AJCC, n (%)				< 0.001
I–II	320 (27)	309 (30)	11 (8)	
IV	861 (73)	737 (70)	124 (92)	
AJCC.T, n (%)				< 0.001
T4a	867 (73)	794 (76)	73 (54)	
T4b	314 (27)	252 (24)	62 (46)	
AJCC.N, n (%)				0.099
N0 & Nx	366 (31)	333 (32)	33 (24)	
N1a & N1b & N1NOS	815 (69)	713 (68)	102 (76)	
AJCC.M, n (%)				< 0.001
M0	1073 (91)	976 (93)	97 (72)	
M1	108 (9)	70 (7)	38 (28)	
Grade, n (%)				< 0.001
I–II	301 (25)	274 (26)	27 (20)	
III–IV	128 (11)	65 (6)	63 (47)	
Unknown	752 (64)	707 (68)	45 (33)	
Chemotherapy, n (%)				< 0.001
No	1125 (95)	1031 (99)	94 (70)	
Yes	56 (5)	15 (1)	41 (30)	
Tumor.size, n (%)				< 0.001
~ 30	619 (52)	581 (56)	38 (28)	
30+	562 (48)	465 (44)	97 (72)	
Hist.type, n (%)				0.391
PA	885 (75)	777 (74)	108 (80)	
FVPTC	263 (22)	239 (23)	24 (18)	
Others	33 (3)	30 (3)	3 (2)	
Multiple.primary.tumors, n (%)				0.403
No	971 (82)	864 (83)	107 (79)	
Yes	210 (18)	182 (17)	28 (21)	
Surgery.method, n (%)				0.001
Subtotal_thyroidectomy	25 (2)	16 (2)	9 (7)	
Total_thyroidectomy	1156 (98)	1030(98)	126 (93)

In the univariate and multivariate analyses, the results of Cox regression showed worse OS (univariate: hazard ratio [HR], 6.45; 95% confidence interval [CI], 4.65–8.94; multivariate: HR, 2.19; 95% CI 1.44–3.34) and DSS (univariate: HR, 8.85; 95% CI 5.77–13.58; multivariate: HR, 2.19; 95% CI 1.44–3.34) for patients who received EBRT. As shown in Table 2, older patients, male patients, widowed patients or those with other marital status, and patients with AJCC M1 stage, grade III–IV stage, chemotherapy, tumor size > 30 mm, or multiple primary tumors had worse OS. Except widowed patients or those with other marital status, the same variable suggested a worse DSS (Table 3).

**Table 2:** Cox regression: univariate and multivariate analyses of overall survival.

	Univariate analysis		Multivariate analysis	
	HR (95% CI)	P value	HR (95% CI)	P value
Age.cat: 51–65 vs. ~ 50	5.205 (2.764–9.8)	< 0.001	3.886 (1.524–9.908)	0.004
Age.cat: 66~ vs. ~ 50	11.688 (6.407–21.323)	< 0.001	6.571 (2.602–16.596)	< 0.001
Sex: male vs. female	1.642 (1.197–2.253)	0.002	1.55 (1.092–2.201)	0.014
Race: black vs. white	0.865 (0.404–1.849)	0.707	0.928 (0.426–2.021)	0.851
Race: others vs. white	0.421 (0.238–0.745)	0.003	0.586 (0.327–1.05)	0.072
Marital_status: divorced/separated vs. married	1.273 (0.735–2.203)	0.389	1.72 (0.972–3.045)	0.063
Marital_status: single/unmarried vs. married	0.79 (0.499–1.252)	0.316	1.307 (0.804–2.126)	0.28
Marital_status: widowed/others vs. married	1.991 (1.313–3.019)	0.001	1.959 (1.223–3.139)	0.005
Diagnosis: 2011–2012 vs. 2010	0.894 (0.593–1.347)	0.591	0.781 (0.514–1.186)	0.246
Diagnosis: 2013–2014 vs. 2010	1.204 (0.77–1.884)	0.416	1.032 (0.651–1.637)	0.894
Diagnosis: 2015 vs. 2010	0.872 (0.421–1.804)	0.711	0.737 (0.355–1.533)	0.415
AJCC: IV vs. I–II	8.536 (3.998–18.222)	< 0.001	1.179 (0.365–3.808)	0.783
AJCC.T: T4b vs. T4a	2.255 (1.637–3.105)	< 0.001	1.417 (0.986–2.035)	0.059
AJCC.N: N1a & N1b & N1NOS vs. N0 & Nx	1.107 (0.782–1.567)	0.566	1.229 (0.843–1.792)	0.284
AJCC.M: M1 vs. M0	3.796 (2.631–5.477)	< 0.001	1.663 (1.107–2.499)	0.014
Grade: III–IV vs. I–II	6.446 (3.999–10.39)	< 0.001	2.153 (1.219–3.804)	0.008
Grade: unknown vs. I–II	1.282 (0.816–2.013)	0.28	1.118 (0.705–1.774)	0.635
Chemotherapy: yes vs. no	8.842 (5.914–13.22)	< 0.001	2.424 (1.395–4.212)	0.002
Tumor.size: 30+ vs. ~ 30	2.672 (1.896–3.766)	< 0.001	1.679 (1.155–2.439)	0.007
Hist.type: FVPTC vs. PA	1.033 (0.705–1.514)	0.868	1.047 (0.706–1.553)	0.82
Hist.type: others vs. PA	0.586 (0.186–1.845)	0.361	0.549 (0.169–1.78)	0.318
Multiple.primary.tumors: yes vs. no	2.153 (1.531–3.028)	< 0.001	1.492 (1.033–2.155)	0.033
Surgery.method: Total_thyroidectomy vs. Subtotal_thyroidectomy	0.424 (0.187–0.959)	0.039	0.527 (0.224–1.238)	0.142
Radiation.type: yes vs. no	6.446 (4.649–8.938)	< 0.001	2.191 (1.438–3.34)	< 0.001

Age: age of diagnosis, Sex: sex, Race: ethnic group, Marital status: marital status, Diagnosis: time of diagnosis, AJCC: AJCC total stage, AJCC T: AJCC T stage, AJCC N: AJCC N stage, AJCC M: AJCC M stage, Grade: pathological grade (Grade I: well differentiated; Grade II: moderately differentiated; Grade III: poorly differentiated; Grade IV: undifferentiated, anaplastic), Chemotherapy: whether chemotherapy, Tumor.size: ≤ 30 mm vs > 30 mm, Hist.type: histological type (PA: papillary adenocarcinoma, Original ICD code: 8260; FVPTC: papillary carcinoma, follicular variant, original ICD code: 8340), Multiple.primary.tumors: whether these are multiple tumors, Surgery.method: surgical method, Radiation type: have or not EBRT(RAI + EBRT&alone EBRT vs alone RAI).

**Table 3:** Cox regression: univariate analysis and multivariate analysis of disease-specific survival.

	Univariate analysis		Multivariate analysis	
	HR (95% CI)	P value	HR (95% CI)	P value
Age.cat: 51–65 vs. ~ 50	6.705 (2.797–16.076)	< 0.001	3.886 (1.524–9.908)	0.004
Age.cat: 66~ vs. ~ 50	11.875 (5.08–27.761)	< 0.001	6.571 (2.602–16.596)	< 0.001
Sex: male vs. female	1.698 (1.109–2.598)	0.015	1.55 (1.092–2.201)	0.014
Race: black vs. white	1.136 (0.459–2.811)	0.783	0.928 (0.426–2.021)	0.851
Race: others vs. white	0.416 (0.191–0.902)	0.026	0.586 (0.327–1.05)	0.072
Marital_status: divorced/separated vs. married	1.687 (0.9–3.161)	0.103	1.72 (0.972–3.045)	0.063
Marital_status: single/unmarried vs. married	0.571 (0.29–1.123)	0.104	1.307 (0.804–2.126)	0.28
Marital_status: widowed/others vs. married	1.205 (0.628–2.311)	0.575	1.959 (1.223–3.139)	0.005
Diagnosis: 2011–2012 vs. 2010	0.875 (0.509–1.504)	0.629	0.781 (0.514–1.186)	0.246
Diagnosis: 2013–2014 vs. 2010	1.034 (0.567–1.883)	0.914	1.032 (0.651–1.637)	0.894
Diagnosis: 2015 vs. 2010	0.648 (0.237–1.768)	0.396	0.737 (0.355–1.533)	0.415
AJCC: IV vs. I–II	16.806 (4.133–68.333)	< 0.001	1.179 (0.365–3.808)	0.783
AJCC.T: T4b vs. T4a	2.725 (1.779–4.173)	< 0.001	1.417 (0.986–2.035)	0.059
AJCC.N: N1a & N1b & N1NOS vs. N0 & Nx	1.23 (0.762–1.985)	0.397	1.229 (0.843–1.792)	0.284
AJCC.M: M1 vs. M0	5.326 (3.372–8.412)	< 0.001	1.663 (1.107–2.499)	0.014
Grade: III–IV vs. I–II	7.83 (4.221–14.528)	< 0.001	2.153 (1.219–3.804)	0.008
Grade: unknown vs. I–II	1.036 (0.557–1.926)	0.91	1.118 (0.705–1.774)	0.635
Chemotherapy: yes vs. no	13.887 (8.608–22.404)	< 0.001	2.424 (1.395–4.212)	0.002
Tumor.size: 30+ vs. ~ 30	4.066 (2.441–6.775)	< 0.001	1.679 (1.155–2.439)	0.007
Hist.type: FVPTC vs. PA	0.77 (0.44–1.346)	0.358	1.047 (0.706–1.553)	0.82
Hist.type: Others vs. PA	0.334 (0.046–2.405)	0.276	0.549 (0.169–1.78)	0.318
Multiple.primary.tumors: yes vs. no	0.234 (0.086–0.639)	0.005	1.492 (1.033–2.155)	0.033
Surgery.Method: Total_thyroidectomy vs. Subtotal_thyroidectomy	0.351 (0.128–0.957)	0.041	0.527 (0.224–1.238)	0.142
Radiation.type: yes vs. no	8.85 (5.768–13.577)	< 0.001	2.191 (1.438–3.34)	< 0.001

In the triclassification variables, the univariate analysis of DSS showed that patients receiving EBRT alone compared RAI alone had an HR of 10.98 (95% CI 7.07–17.04) (P < 0.001). The HR value for the multivariate analysis of DSS was 2.743 (95% CI 1.74–4.32) (P < 0.001). Statistical differences showed that patients receiving EBRT alone had worse DSS than those receiving RAI alone, while patients receiving RAI did not show survival difference compared to patients receiving concurrent EBRT. Similar conclusions can be obtained in the univariate and multivariate analyses of OS.

### Cumulative incidence analysis

**Figure 2:**
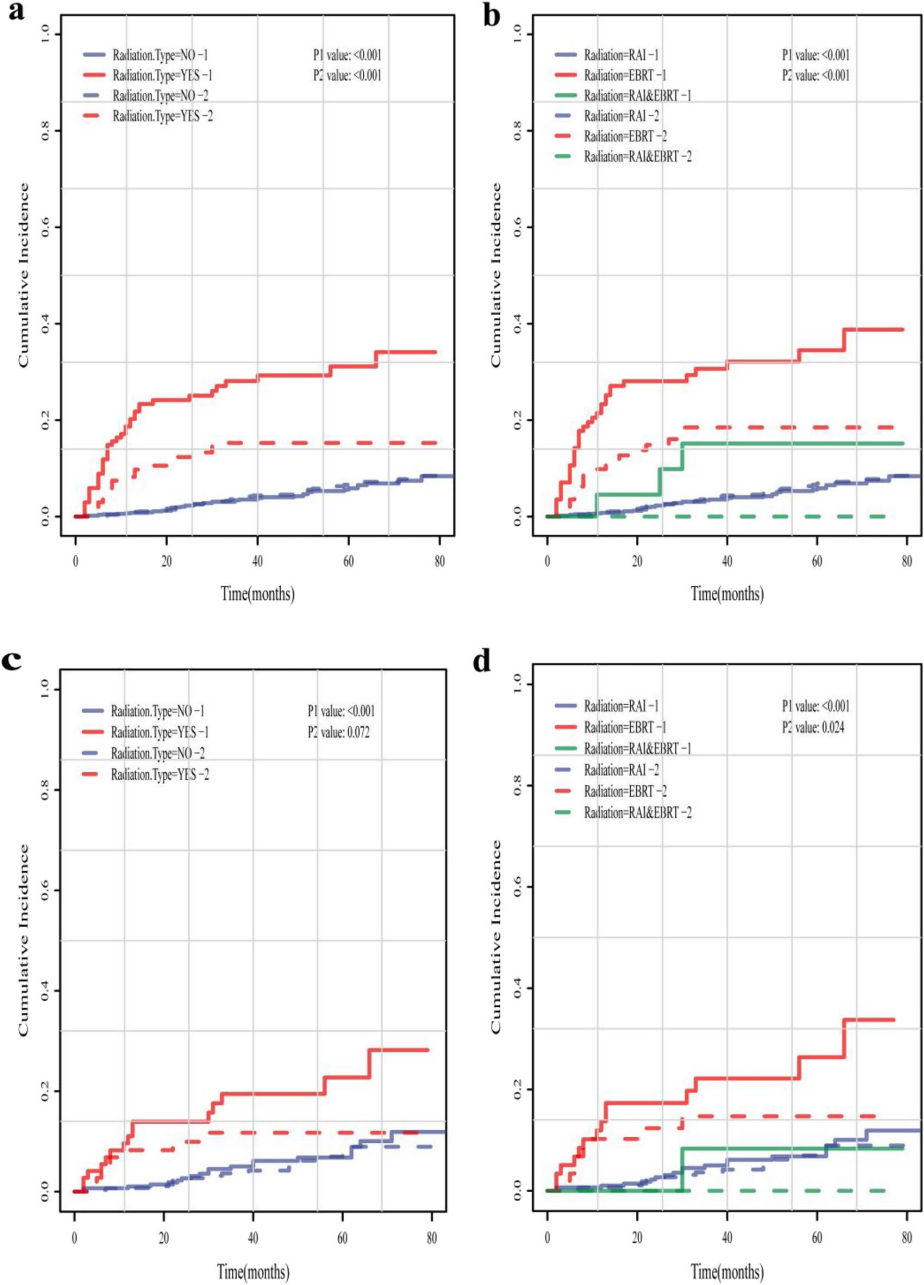
(**a**) Before propensity score-matched: cumulative incidence of the dichotomy variables. (**b**) Before propensity score-matched: cumulative incidence of the trichotomy variables. (**c**) After propensity score-matched: cumulative incidence of the dichotomy variables. (**d**) After propensity score-matched: cumulative incidence of the trichotomy variables [P1 is the P value of outcome = 1 (death of thyroid cancer); P2 is the P value of outcome = 2 (death not caused by thyroid cancer, competition event)].

As shown above, the P-value of the cumulative risk curve for accepting the EBRT dichotomy variables (Fig. 2a) and trichotomy variables (Fig. 2b) was statistically significant, indicating that, in patients with locally invasive thyroid cancer who underwent total or subtotal resection surgery, patients undergoing EBRT had a higher cumulative risk of death from thyroid cancer. The other variables that led to a higher cumulative risk of death from thyroid cancer were as follows: older age, AJCC stage IV, AJCC T4b stage, AJCC M1 stage, chemotherapy, grade III–IV stage, tumor size > 30 mm, no multiple primary tumors, and male sex.

### Competitive risk model analysis

**Figure 3:**
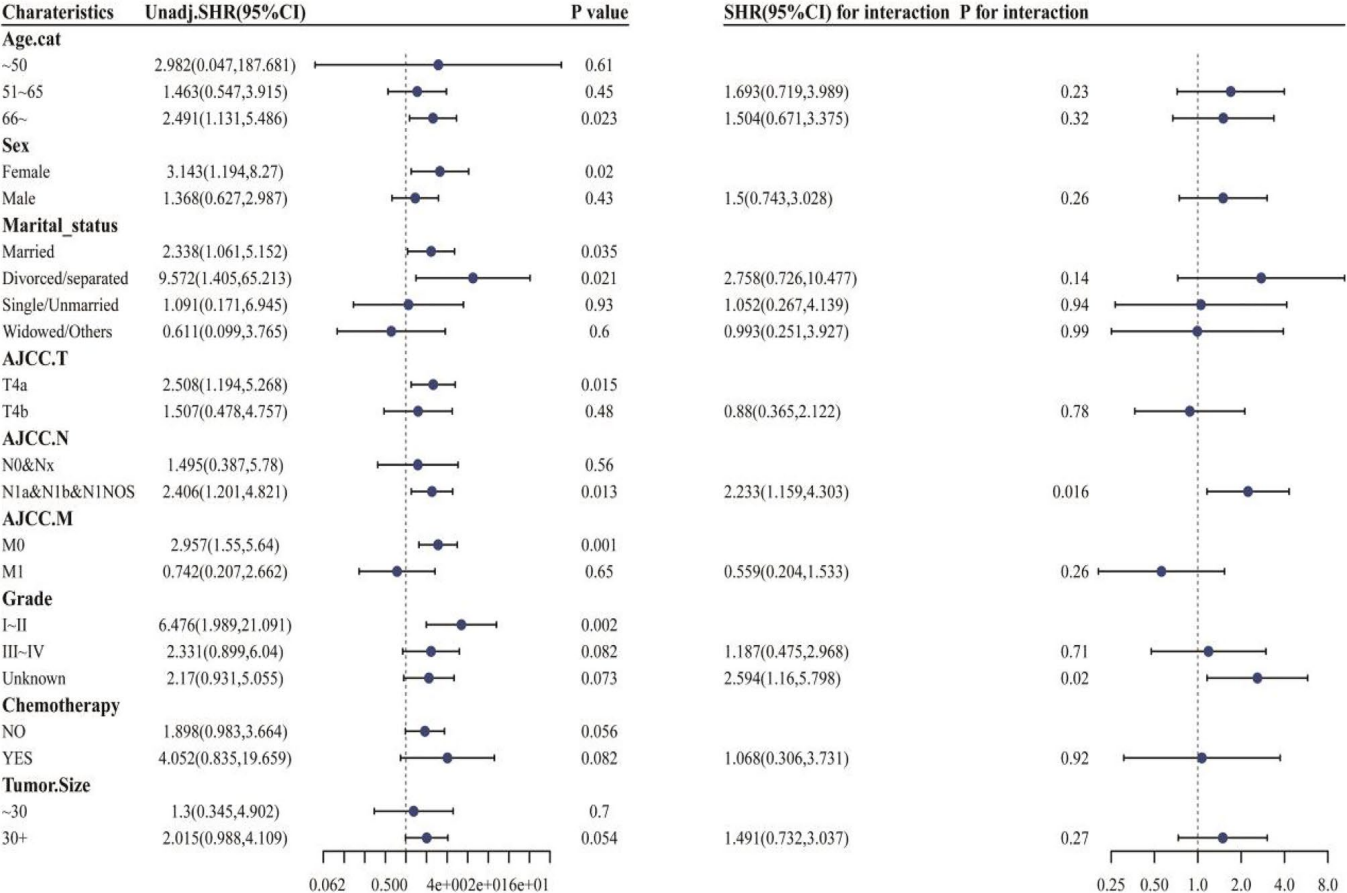
Subgroup analysis based on competitive risk model (multivariate).

Competitive events of non-cancer death exist in patients with cancer, such as cardiac death, suicide, and cerebrovascular accidents. The Cox proportional hazards model can overestimate the incidence of outcome events, and the competing risk model can more accurately assess the association between predictor variables and outcome events. We conducted a subgroup analysis of patients receiving EBRT to determine whether there were treatment-associated factors associated with death from thyroid cancer. In the N1 subgroup, EBRT was a risk factor in disease-specific death (HR, 2.406, P = 0.013). A significant interaction of the N1 subgroup with the “association of EBRT with disease-specific death” was observed (P_interaction_ = 0.016) (Fig. 3). In the tricategorical variables competing risk model, patients who received EBRT alone had a higher risk of death from thyroid cancer than those who received RAI alone. The SHR for the univariate analysis was 9.301 (95% CI 5.99–14.44) (P < 0.01), and the SHR for the multivariate analysis was 1.97 (95% CI 1.03–3.78) (P = 0.042) (Table 4).

**Table 4:** Univariate and multivariate analyses of the competitive risk model under three classifications.

	Univariate analysis		Multivariate analysis	
	SHR (95% CI)	P value	SHR (95% CI)	P value
Age.cat: 51–65 vs. ~ 50	6.536 (2.727–15.667)	< 0.001	4.168 (1.31–13.264)	0.016
Age.cat: 66~ vs. ~ 50	10.947 (4.68–25.605)	< 0.001	7.221 (2.304–22.631)	< 0.001
Sex: male vs. female	1.661 (1.087–2.538)	0.019	1.956 (1.139–3.361)	0.015
Race: black vs. white	1.158 (0.468–2.863)	0.75	1.652 (0.577–4.726)	0.35
Race: others vs. white	0.425 (0.196–0.922)	0.03	0.676 (0.332–1.378)	0.28
Marital_status: divorced/separated vs. married	1.695 (0.912–3.15)	0.095	2.236 (1.069–4.676)	0.033
Marital_status: single/unmarried vs. married	0.57 (0.292–1.116)	0.1	0.924 (0.454–1.882)	0.83
Marital_status: widowed/others vs. married	1.14 (0.593–2.19)	0.69	1.367 (0.636–2.939)	0.42
Diagnosis: 2011–2012 vs. 2010	0.857 (0.507–1.449)	0.56	0.744 (0.392–1.413)	0.37
Diagnosis: 2013–2014 vs. 2010	0.98 (0.549–1.751)	0.95	0.897 (0.488–1.649)	0.73
Diagnosis: 2015 vs. 2010	0.608 (0.226–1.635)	0.32	0.41 (0.158–1.07)	0.068
AJCC: IV vs. I–II	16.131 (3.972–65.517)	< 0.001	2.26 (0.388–13.172)	0.36
AJCC.T: T4b vs. T4a	2.644 (1.73–4.04)	< 0.001	1.303 (0.741–2.292)	0.36
AJCC.N: N1a & N1b & N1NOS vs. N0 & Nx	1.224 (0.761–1.969)	0.4	1.223 (0.712–2.099)	0.47
AJCC.M: M1 vs. M0	5.066 (3.21–7.994)	< 0.001	1.891 (1.033–3.46)	0.039
Grade: III–IV vs. I–II	7.1 (3.824–13.182)	< 0.001	2.216 (0.929–5.287)	0.073
Grade: unknown vs. I–II	1.022 (0.552–1.893)	0.94	0.9 (0.466–1.739)	0.75
Chemotherapy: yes vs. no	12.134 (7.338–20.064)	< 0.001	3.564 (1.463–8.679)	0.005
Tumor.size: 30+ vs. ~ 30	3.971 (2.384–6.616)	< 0.001	1.903 (1.066–3.398)	0.03
Hist.type: FVPTC vs. PA	0.764 (0.437–1.333)	0.34	0.664 (0.318–1.387)	0.28
Hist.type: others vs. PA	0.338 (0.046–2.506)	0.29	0.252 (0.024–2.606)	0.25
Multiple.primary.tumors: yes vs. no	0.211 (0.077–0.581)	0.003	0.105 (0.037–0.301)	< 0.001
Surgery.method: Total_thyroidectomy vs. Subtotal_thyroidectomy	0.364 (0.125–1.062)	0.064	0.444 (0.125–1.578)	0.21
Radiation: EBRT vs. RAI	9.301 (5.993–14.435)	< 0.001	1.97 (1.026–3.78)	0.042
Radiation: RAI & EBRT vs. RAI	2.798 (0.864–9.057)	0.086	1.291 (0.362–4.605)	0.69

### After PSM analysis

This study used PSM to control for the influence of confounding factors on the study conclusions. PSM yielded 365 patients for analysis:73 in the EBRT group and 292 in the non-EBRT group. Except for AJCC M, grade stage, and whether they received chemotherapy, these variables were evenly distributed between the groups after PSM (Table 5).

**Table 5:** Patient characteristics after PSM.

Variables	Total (n = 365)	No (n = 292)	Yes (n = 73)	p
Age.cat, n (%)				0.729
~ 50	95 (26)	78 (27)	17 (23)	
51–65	146 (40)	114 (39)	32 (44)	
66~	124 (34)	100 (34)	24 (33)	
Sex, n (%)				0.807
Female	232 (64)	187 (64)	45 (62)	
Male	133 (36)	105 (36)	28 (38)	
AJCC, n (%)				0.912
I–II	54 (15)	44 (15)	10 (14)	
IV	311 (85)	248 (85)	63 (86)	
AJCC.T, n (%)				0.793
T4a	263 (72)	209 (72)	54 (74)	
T4b	102 (28)	83 (28)	19 (26)	
AJCC.N, n (%)				0.888
N0 & Nx	115 (32)	93 (32)	22 (30)	
N1a & N1b & N1NOS	250 (68)	199 (68)	51 (70)	
AJCC.M, n (%)				0.002
M0	324 (89)	267 (91)	57 (78)	
M1	41 (11)	25 (9)	16 (22)	
Grade, n (%)				< 0.001
I–II	94 (26)	76 (26)	18 (25)	
III–IV	52 (14)	24 (8)	28 (38)	
Unknown	219 (60)	192 (66)	27 (37)	
Chemotherapy, n (%)				< 0.001
No	342 (94)	287 (98)	55 (75)	
Yes	23 (6)	5 (2)	18 (25)	
Tumor.size, n (%)				1
~ 30	179 (49)	143 (49)	36 (49)	
30+	186 (51)	149 (51)	37 (51)	
Hist.type, n (%)				0.759
PA	270 (74)	214 (73)	56 (77)	
FVPTC	91 (25)	74 (25)	17 (23)	
Others	4 (1)	4 (1)	0 (0)	
Multiple.primary.tumors, n (%)				0.247
No	304 (83)	247 (85)	57 (78)	
Yes	61 (17)	45 (15)	16 (22)	
Surgery.method, n (%)				0.588
Subtotal_thyroidectomy	5 (1)	5 (2)	0 (0)	
Total_thyroidectomy	360 (99)	287 (98)	73(100)	

In the propensity score-matched Kaplan–Meier analysis, patients who received EBRT still had worse OS (6-year OS, 59.62% vs 74.6%; P < 0.001) (Fig. 1c) and DSS (6-year DSS, 66.6% vs 78.2%; P < 0.001) (Fig. 1d) than patients who did not receive EBRT. Therefore, patients who received EBRT had worse survival than those who did not receive EBRT.

After PSM, the P1 values obtained using the cumulative risk curves of the dichotomous classification (Fig. 2c) and tricategorical variables (Fig. 2d) were statistically significant (P < 0.001). Furthermore, patients who received EBRT had a higher cumulative risk of death from thyroid cancer after PSM.

### Sensitivity analysis

Given the possibility of unmeasured confounders, sensitivity analysis can further assess the robustness of the results of the primary variable analysis. The E value for receiving EBRT was equal to 1. Therefore, there was no need to evaluate the effects of unknown confounders. In the sensitivity analysis of the three categorized variables, E = 3.52 is shown for the EBRT vs RAI and E > HR (HR = 1.97), so the results are robust. The E-value for RAI and EBRT vs. RAI = 1, and the effects of unknown confounding factors does not need to be assessed.

## Discussion

This study investigated the prognostic value of adjuvant external irradiation in adult patients with locally invasive thyroid cancer based on competing risk models and PSM. The main conclusions are as follows: through Cox regression and competing risk models, receiving EBRT is a poor prognostic factor affecting OS and thyroid cancer-specific death in adult patients with locally invasive thyroid cancer. Both before and after PSM, patients receiving EBRT had a higher cumulative risk of death from thyroid cancer than those in the non-recipient group (P < 0.001). We performed a subgroup analysis of patients undergoing EBRT and found a significant interaction between patients receiving EBRT with lymph node metastases and disease-specific death.

OS is a controversial area in the current literature. The majority of published article results showed that there was no statistically significant difference in OS in patients who underwent EBRT compared to those who did not^17^. Keum et al. showed that, in patients who received EBRT compared to those who did not, 5- and 10-year OS rates were 76% vs 81.4% and 63% vs 79%, respectively^13^. The results of a study by Lee et al. showed that the 5- and 10-year OS rates were 96.9% vs 99.2% and 86.4% vs 93.5%, respectively^18^. Although the results were not statistically significant, patients receiving EBRT showed a trend toward worse OS with a longer follow-up time. The results of this study showed that the OS was 57.8% vs 91.6% for patients who received EBRT before PSM and 69.9% vs 89.7% after PSM, both were statistically significant (P < 0.001). A 405-population-based study showed that, targeting well-differentiated thyroid cancers with pT4 stage, the 10- and 15-year OS rates were 85.7% and 77.1%, respectively, for the non-EBRT group and 67.5% and 57.0%, respectively, for the EBRT group (HR, 2.229; 95% CI 1.570–3.164; P < 0.0001)^19^. A study by Megwalu et al. similarly confirmed that EBRT was associated with worse overall survival^20^. The reason for the difference in survival results may be related to the small sample size of previous studies, which was not sufficient to detect survival differences. Meanwhile, because differentiated thyroid cancer is an indolent disease, a longer follow-up of more patients can provide more accurate response results.

The study similarly confirmed that EBRT is associated with worse DSS. After PSM, patients who received EBRT had a statistically significant DSS of 79.5% vs 93.5% rather than those who did not receive it. The study by Sit et al. showed that the 10- and 15-year DSS were 84.1% and 80.2%, respectively, for the non-EBRT group and 93.1% and 90.8%, respectively, for the EBRT group (HR, 2.102; 95% CI 1.219–3.623; P = 0.0075). EBRT was not significantly associated with CSS^19^. Contrary to this study, Chow et al. aimed at patients with gross and local residual disease after surgery and found that receiving EBRT improved DSS for 10 years (74.1% vs. 49.7%; P = 0.01)^21^. Brieley et al. reported that, in patients with stage T4 aged > 60 years with extrathyroid expansion without gross residual disease, a higher CSS (10-year CSS 81% vs 64·6%; P = 0·04) was noted in patients who received RT than in those who did not^11^. The reasons for these conflicting results may be the heterogeneity in the inclusion criteria of the study population.

The cumulative risk curve of the study was performed using EBRT as the main variable. Before and after PSM, patients receiving EBRT had a higher risk of death from thyroid cancer than those who did not (P < 0.001). Besides EBRT, other adverse prognostic factors for thyroid cancer death after PSM were age, AJCC IV stage, chemotherapy, grade III to IV stage, and tumor size > 30 mm. According to the latest ESMO clinical practice guidelines for thyroid cancer, the most important prognostic factors for thyroid cancer include tissue type, primary tumor size, extraglandular invasion, and vascular invasion^22,23^. These two conclusions have partially similar prognostic factors, which need to be further demonstrated in future studies.

Even in locally advanced, well-differentiated thyroid cancer cases, the 10-year survival rate is close to 80% to 90%, and a large proportion of these deaths can be attributed to other causes. To accurately assess the association between EBRT and thyroid cancer death, this study performed a univariate and multivariate analyses, and a subgroup analysis under a competing risk model. The results of the univariate and multivariate analyses showed that patients receiving EBRT alone had a higher risk of death from thyroid cancer than RAI alone. We performed a subgroup analysis of patients receiving EBRT to determine whether self- or treatment-associated factors were associated with death from thyroid cancer. Only lymph node metastases were significantly associated with thyroid cancer death (P = 0.016). The American Thyroid Association guidelines for the management of adult thyroid nodules and differentiated thyroid cancer state that neck dissection is performed in patients with well-differentiated thyroid cancer if there are clinically positive lymph nodes^16^. The American Head and Neck Society does not recommend routine adjuvant EBRT for cervical lymph node metastases^24^.

Although this study provides a comprehensive analysis of the clinical characteristics and prognostic factors of patients with PTC, some limitations remain: the major limitation of this study is that patients who underwent EBRT had many risk factors of poor survival, and the PSM method did not make perfect matching of these variables: more patients with distant metastasis and high-grade tumor included in the EBRT group even after matching, and more patients underwent chemotherapy in this group. Second, as a population-based study in the SEER database^25^, some important data related to patient outcomes, such as positive margins, residual disease, chemotherapy regimen, and dose, are missing, which often affect the decision to use EBRT for treatment. Moreover, the SEER database did not include data on EBRT related side effects. Third, the follow-up outcome of the SEER database included only death and its cause (i.e., OS and CSS), which has limitations on study recurrence, metastasis, or progression. Finally, with continuous improvements in disease pathogenesis, such as the administration of targeted drugs and immunotherapy, the prognosis of patients will certainly change^26,27^. Given the limitations of this study, the findings should be interpreted with caution.

In conclusion, patients with locally advanced PTC have a good prognosis. The results of this study suggest that the addition of EBRT to thyroidectomy and RAI as part of the initial treatment regimen for patients with locally invasive PTC does not provide a survival benefit.

## Methods

Patients were recruited from the SEER Program of the United States National Cancer Institute, which was released in April 2020. This study retrospectively analyzed data from the SEER database from 2010 to 2015. Patients’ demographic and clinical data were downloaded using the SEER*Stat software. The data are public and do not involve the privacy of patients, therefore, review and consent of the ethics committee are not required. We identified 1181 patients with locally invasive T4 PTC from January 1, 2010 to December 31, 2015, for the initial analysis. The following International Classification of Diseases for Oncology (ICD-O) code was used: C73.9 for thyroid gland. The following ICD-O-3 histology codes for papillary carcinoma were included: 8050/3, papillary carcinoma, NOS; 8260/3, papillary adenocarcinoma, NOS; 8340/3, papillary carcinoma, follicular variant; 8342/3, papillary carcinoma, oxyphilic cell; and 8343/3, papillary carcinoma, encapsulated. The inclusion criteria were treatment with total/near-total thyroidectomy (with or without neck dissection), age ≥ 18 years, and radiation recode of 1, 2, or 8 (corresponding to RAI + EBRT, EBRT, or RAI alone). Patients who do have definite survival months, and SEER cause-specific death classification and those who did not undergo total/near-total thyroidectomy were excluded from the study. Two treatment groups were identified: patients who received EBRT and those who did not. Concurrently, three treatment groups were identified: patients who received RAI + EBRT, those who received EBRT alone, and those who received RAI alone.

A detailed definition of all variables in this study is available in the SEER database manual. The following variables were included in the multivariate analysis for adjustment: age, sex, race , marital status, time of diagnosis (Diagnosis), AJCC total stage, AJCC T stage, AJCC N stage, AJCC M stage, pathological grade (Grade), chemotherapy, tumor size, histological type, whether these are multiple tumors (Multiple.Primary.Tumors) and surgery method. The classifications of these variables are defined as: Age is a continuous variable, according to the tertile, converted to trichotomy: ~ 50 vs 51–65 vs 66~; Sex is a dichotomous variable: Male vs Female; Race converted multiple classification variables into trichotomy: White vs Black vs Others; Marital status converted to four categories: Single/Unmarried vs Married vs Divorced/Separated vs Widowed/ Others; Diagnosis is the time period of case inclusion and converted into four categories:2010 vs 2011–2012 vs 2013–2014 vs 2015;AJCC is converted to dichotomy: I–II vs IV; AJCC T Stage is converted to dichotomy: T4a vs T4b; AJCC N stage is converted to dichotomy: N0 & Nx vs N1; AJCC M Stage is converted to dichotomy: M0 vs M1;Grade is converted to trichotomy: I–II vs III–IV vs Unknown;Chemotherapy is a dichotomous variable: Yes vs No; Tumor size as a continuous variable, take with survMisc package the boundary value Cut-off = 33 and take the integer to convert into two classification:30 mm vs > 30 mm; Hist Type is converted to trichotomy: 8260 (PA) vs 8340 (FVPTC) vs others; Multiple Primary Tumors is a dichotomous variable: Yes vs No; Surgery Method two classification: total thyroidectomy vs Subtotal thyroidectomy. The primary outcome variable was disease-specific cumulative survival (DSS). The secondary outcome variable was overall survival (OS). The primary endpoint used for comparison was cause-specific survival (CSS). We selected CSS as the outcome of interest, whereas death due to other causes was considered a competing risk event, and a patient who was alive was regarded as a censored event.

In addition, we defined the predicted probability of treatment as a propensity score (PS) to balance the clinicopathologic factors between the EBRT and non-EBRT groups in the SEER cohort using the following baseline characteristics that were strongly related to survival but less strongly related to the treatment: age, sex, race, marital status, American Joint Committee on Cancer (AJCC) total stage, AJCC T stage, AJCC N stage, tumor size, histologic type, multiple primary tumors, and surgical method. We performed 1:4 PSM for patients who received or did not receive EBRT, and their prognoses were also compared. The PS is calculated using logit model. The PS for each patient was obtained using a logistic Cox regression model based on patient characteristics. We selected a caliper value at 10% of the standard deviation of the PS value converted by the logit model because it is commonly used. For the competing risk model, we constructed a model as described in a previous study. R (version 4.1.1, https://www.r-project.org/) was used for the statistical analysis, and a two-sided A P-value of < 0.05 was considered statistically significant.

## Data Availability

The data that support the findings of this study are available from the corresponding author upon reasonable request.
